# Biosynthesis of Spatially Differentiated Cellulose for Flexible All‐in‐one Supercapacitors

**DOI:** 10.1002/advs.77603

**Published:** 2026-09-27

**Authors:** Xin Shu, Yi Lu, Xuetong Shi, Zhangmin Wan, Peijin Jiang, Minke Yang, Qingshi Tu, Orlando J. Rojas

**Affiliations:** ^1^ Department of Chemical and Biological Engineering The University of British Columbia Vancouver British Columbia Canada; ^2^ Bioproducts Institute The University of British Columbia Vancouver British Columbia Canada; ^3^ Institute of Process Engineering Chinese Academy of Sciences Beijing PR China; ^4^ Department of Wood Science The University of British Columbia Vancouver British Columbia Canada; ^5^ Department of Chemistry The University of British Columbia Vancouver British Columbia Canada

**Keywords:** all‐in‐one supercapacitor, bacterial cellulose, biosynthesis, spatial differentiation, structural continuity

## Abstract

Bacterial cellulose (BC) is a sustainable scaffold for flexible energy‐storage devices, but conventional biosynthesis provides limited control over through‐thickness structure, making it difficult to integrate distinct functional regions within a monolithic structure. Here, we use oxygen‐guided biosynthesis to grow spatially differentiated BC scaffolds (SDBC) for flexible all‐in‐one supercapacitors. By creating air‐water and water‐oil oxygen‐accessible interfaces, the microbes’ aerobic nature directs cellulose deposition into two dense biofilms connected by an open fibrillar interlayer in a single cultivation step. The dense surface region supports polyaniline loading and charge storage, while the interlayer accommodates the gel electrolyte and facilitates ion transport. The resulting supercapacitor combines structural continuity with deformation tolerance, achieving high ionic conductivity (112.0 mS cm^−1^), mechanical toughness (1.77 MJ m^−3^), areal capacitance (410.6 mF cm^−2^), and energy density (34.0 µWh cm^−2^). This work establishes a bottom‐up route to introduce spatial differentiation and structural continuity during cellulose biosynthesis and provides a platform for monolithic soft energy‐storage devices.

## Graphical abstract

Spatially differentiated bacterial cellulose is grown by oxygen‐guided biosynthesis to form dense surface regions connected by an open fibrillar interlayer. This architecture supports electroactive material loading, ion transport, and mechanical stability in an all‐in‐one supercapacitor, showing how biological assembly can create device‐ready structures without conventional lamination.

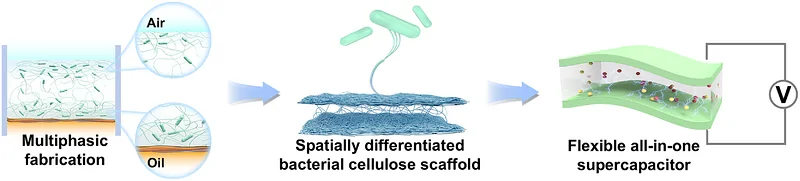

## 1 Introduction

Flexible all‐in‐one supercapacitors (SCs) are attractive energy‐storage systems for wearable electronics, soft robotics, and portable devices because they can combine charge storage, mechanical compliance, and structural integration within a single monolithic construct [1, 2, 3, 4, 5]. However, their performance depends critically on how the electrode and electrolyte domains are organized. Efficient devices require electroactive regions with high active‐material loading and stable electron transport and charge storage, while also maintaining ion‐conductive pathways that allow rapid electrolyte transport under mechanical deformation [6, 7]. In conventional multilayer or post‐assembled supercapacitors, these requirements are often difficult to satisfy simultaneously because the interfaces between electrode and electrolyte layers can introduce contact resistance, structural discontinuities, and delamination during bending, compression, or twisting [8, 9, 10, 11].

A promising strategy is to construct devices from scaffolds that are spatially differentiated, so that different regions of the same continuous material can perform distinct functions [7]. In such configurations, dense surface regions could provide mechanically robust, high‐surface‐area domains for electroactive material deposition [12], whereas a more open internal region could accommodate the electrolyte phase and facilitate through‐thickness ion transport [13, 14]. This type of density‐differentiated architecture is particularly favourable for all‐in‐one supercapacitors, where electroactive and ion‐conductive components remain closely coupled but functionally distinct. Nevertheless, fabricating such structures remains challenging. Conventional lamination, coating, or stacking approaches can generate layered or graded architectures, but they rely on sequential assembly of discrete components, often compromising structural continuity, transport efficiency, and mechanical stability [7, 14].

Bacterial cellulose (BC) offers an appealing scaffold platform for flexible electrochemical devices because of its high crystallinity, mechanical robustness, biocompatibility, and continuous three‐dimensional nanofibrillar network [15, 16, 17, 18, 19]. In plant‐derived cellulose materials, defined structures and functions are commonly introduced after top‐down extraction or modification [5, 20, 21, 22]. By contrast, BC is produced directly by microorganisms as an interconnected hydrogel network [9, 23]. This bottom‐up biosynthetic process provides an opportunity to encode structure during material growth rather than imposing architecture through post‐processing. Previous studies have demonstrated that BC films have been used as separate electrode or electrolyte components in energy storage devices [13, 24, 25, 26, 27]. For example, BC can reinforce PVA/PAA gel electrolyte with weak ionic interactions to achieve an ionic conductivity of 105 mS cm^−1^ and a tensile strength of 0.78 MPa [24]. The porous BC nanofibril network has also served as a scaffold for graphene and polyaniline [27]. Used as the electrode in a post‐assembled symmetric supercapacitor, this composite delivered a specific capacitance of 645 F g^−1^ at 1 A g^−1^. Despite previous efforts, integrating multiple functional regions within a continuous BC scaffold while generating spatial differentiation during biosynthesis remains challenging. This limitation arises in part from the aerobic nature of BC‐producing bacteria, where cellulose biosynthesis is strongly governed by oxygen availability, leading to preferential deposition at oxygen‐rich interfaces [23, 28, 29]. Such oxygen‐confined growth typically yields thin biofilms with limited stratification, restricting spatial control across the thickness of the material [30, 31]. As a result, the direct use of BC biosynthesis to generate device‐specific architectures remains underdeveloped.

**FIGURE 1:**
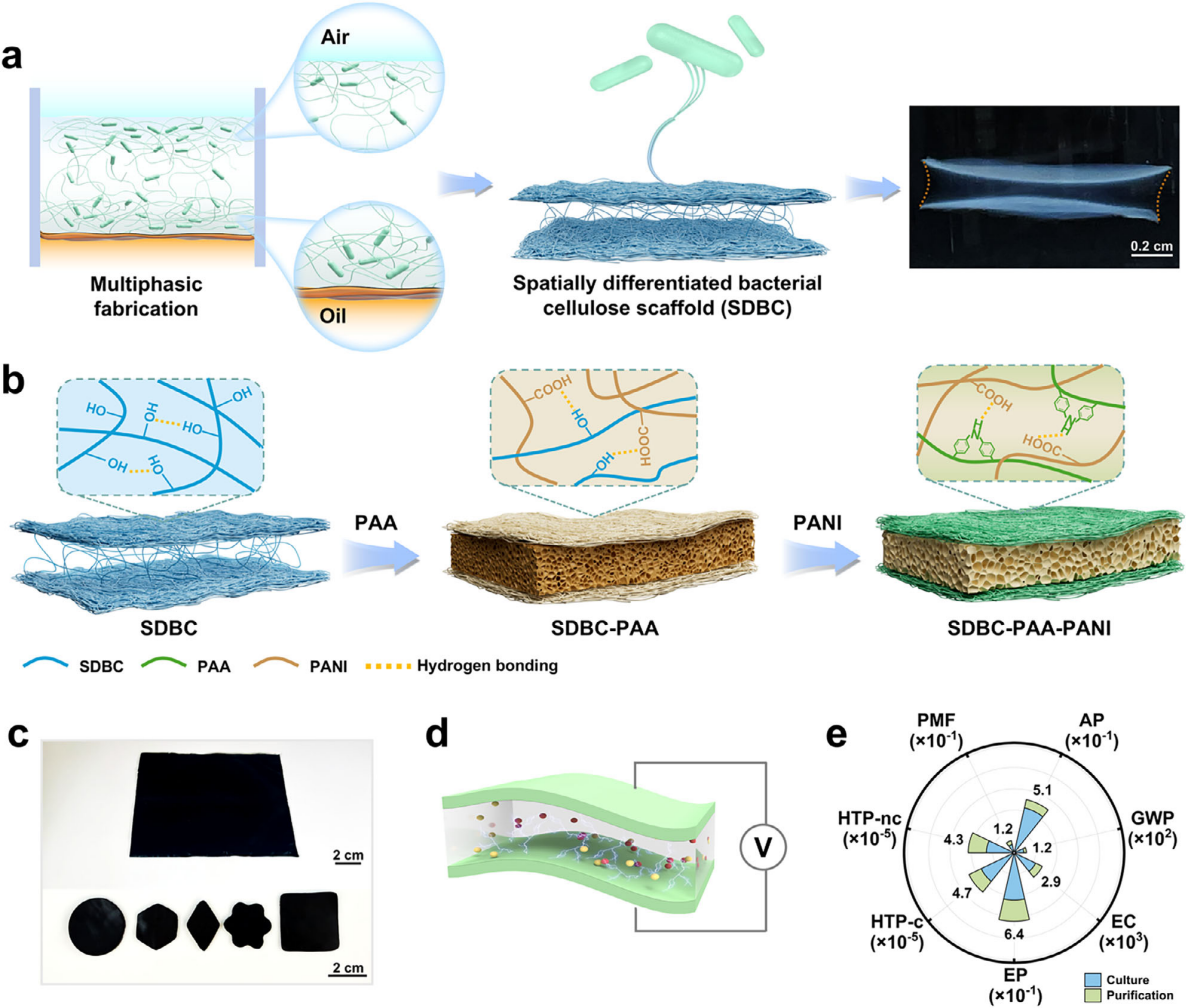
Schematic illustration of the multiphasic fabrication strategy for spatially differentiated bacterial cellulose scaffold (SDBC) and its subsequent functionalization. (a) Multiphasic oxygen‐interface engineering used to direct growth‐encoded spatial differentiation during BC biosynthesis, yielding a monolithic dense‐open‐dense architecture. (b) Sequential incorporation of poly (acrylic acid) (PAA) and polyaniline (PANI) into the SDBC with multiscale coupling. (c) Photographs of SDBC‐PAA‐PANI hydrogels fabricated in different sizes and shapes, demonstrating scalability and shape adaptability. (d) Demonstration of ion transport within the functionalized SDBC. (e) Cradle‐to‐gate life cycle assessment (LCA) of obtaining 1 kg SDBC via the multiphasic fabrication route, suggesting its feasibility and low environmental impact. Impact categories include acidification potential (AP, kg SO_2_‐eq), global warming potential (GWP, kg CO_2_‐eq), freshwater ecotoxicity (EC, CTUe), eutrophication potential (EP, kg N‐eq), human toxicity carcinogenic (HTP‐c, CTUh), human toxicity non‐carcinogenic (HTP‐nc, CTUh), and particulate matter formation (PMF, kg PM_2.5_‐eq).

We address this limitation by using oxygen regulation to grow a spatially differentiated BC scaffold (SDBC). Here, spatial differentiation refers to the emergence of regions with distinct structural density and transport function within a single, continuous biosynthesized scaffold. This outcome is achieved by introducing an oil phase with dissolved oxygen underneath the aqueous culture medium, thereby establishing two oxygen sources at the air‐water (A/W) and water‐oil (W/O) interfaces (Figure 1a). Within this multiphasic environment, the inherent aerobic nature of a high‐yield BC‐producing strain, *Komagataeibacter medellinensis* [23, 30], directs cellulose biosynthesis preferentially toward both parallel interfaces, yielding a dense‐open‐dense BC architecture in a single cultivation step. The resulting SDBC comprises two parallel, mechanically robust and dense biofilms bridged by a fibrillar interlayer that forms out‐of‐plane under reduced oxygen availability, providing structural continuity across the full scaffold thickness. Because SDBC arises from uninterrupted cellulose deposition within a shared biosynthesis history, the interfaces between regions are growth‐defined, imparting structural continuity and strength.

This growth‐defined architecture directly supports the functional requirements of the supercapacitor device. After incorporation of poly(acrylic acid) (PAA) as the gel electrolyte and in situ deposition of polyaniline (PANI) as the electroactive component (Figure 1b), the dense BC‐rich surface regions provide abundant anchoring sites for PANI loading, while the lower‐density internal region accommodates the electrolyte phase and promotes ion transport. The complexation among SDBC and functional parts enables intimate intermolecular coupling with extra mechanical reinforcement and support, which contributes to high mechanical flexibility and dimensional mouldability of the SDBC‐PAA‐PANI architecture (Figure 1c). The SDBC‐PAA‐PANI construct couples ionic transport, charge storage, and mechanical stability within a continuous all‐in‐one supercapacitor architecture (Figure 1d). As a result, the device exhibits high ionic conductivity, mechanical toughness, stable electrochemical performance, and deformation‐tolerant operation.

Overall, this study demonstrates how growth‐defined BC scaffolds can function as integrative platforms for multifunctional systems. By encoding spatial differentiation during oxygen‐guided biosynthesis, this work establishes a route to all‐in‐one supercapacitors in which electroactive loading, ion transport, and mechanical integrity are defined by the biosynthesized scaffold itself, while maintaining a low environmental footprint (Figure 1e).

## 2 Results and Discussion

### 2.1 Single‐Step SDBC Fabrication

**FIGURE 2:**
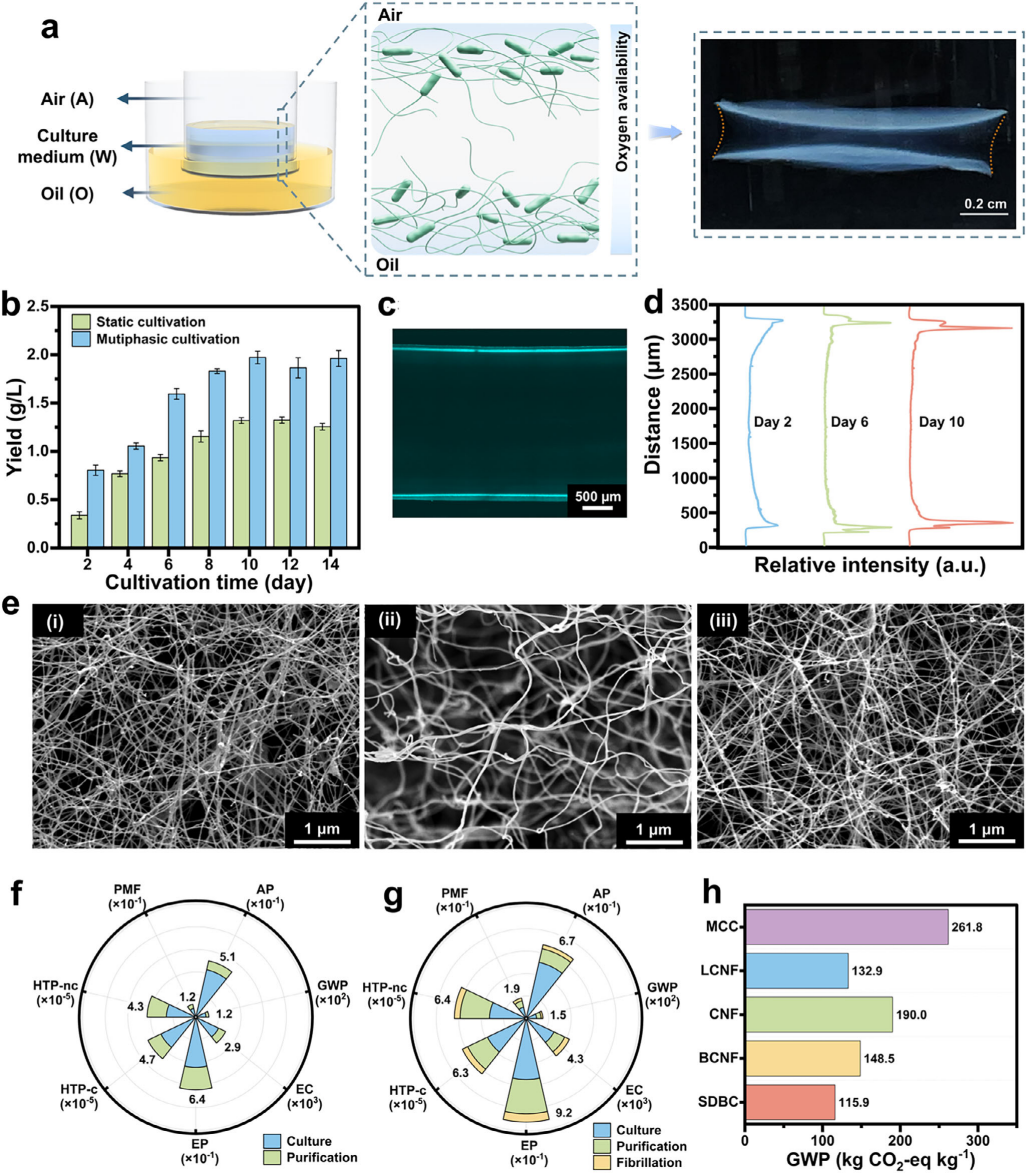
Structural characterization and sustainability implications of SDBC. (a) Schematic illustration of multiphasic bottom‐up biosynthesis of SDBC, showing differentiated cellulose fibril density across the scaffold thickness and a representative side view of the formed architecture. (b) BC yield obtained under conventional single‐interface cultivation and the multiphasic fabrication approach. Data are presented as mean ± SD (*n* = 5). (c) Confocal fluorescence image of the cross‐section of SDBC on cultivation day 10. (d) Fluorescence intensity profiles across SDBC after 2, 6, and 10 days of cultivation. (e) Scanning electron microscopy (SEM) images of SDBC showing the (i) plane‐view of the top biofilm, (ii) cross‐section of the open interlayer formed between the biofilms, and (iii) plane‐view of the bottom biofilm. Cradle‐to‐gate life cycle assessment (LCA) results for obtaining 1 kg SDBC via multiphasic fabrication route (f) and conventional static cultivation and top‐down fibrillation (g). Impact categories include acidification potential (AP, kg SO_2_‐eq), global warming potential (GWP, kg CO_2_‐eq), freshwater ecotoxicity (EC, CTUe), eutrophication potential (EP, kg N‐eq), human toxicity carcinogenic (HTP‐c, CTUh), human toxicity non‐carcinogenic (HTP‐nc, CTUh), and particulate matter formation (PMF, kg PM_2.5_‐eq). (h) Global warming potentials to produce 1 kg SDBC and bacterial cellulose nanofibers (BCNF), together with reported values for wood‐pulp‐derived cellulose nanofibers (CNF) [34], sugarcane bagasse‐derived lignocellulosic nanofibers (LCNF) [35], and microcrystalline cellulose (MCC) [36].

Microbial assembly guided by environmental cues provides a sustainable route to advanced material architectures. Here, we report a single‐step strategy for producing SDBC by engineering a multiphasic biosynthesis environment (Figure 2a). The approach regulates the distribution of cellulose‐producing bacteria (*Komagataeibacter medellinensis*) by establishing two oxygen‐accessible interfaces (A/W and W/O) above and below the culture medium (W) where the oxygen is supplied from the air (A) at the top and from the oxygen‐dissolving hydrofluoroether oil phase (O) at the bottom. This configuration exploits the intrinsic aerobic nature of the bacteria, directing both motility and cellulose biosynthesis to yield a BC framework spanning the full thickness of the culture medium. As in conventional static cultivation, cellulose production increases progressively with cultivation time (Figure 2b). Notably, introducing two biosynthesis interfaces significantly increased the cellulose yield by 54%, from 1.3 ± 0.03 to 2.0 ± 0.07 g L^−1^ (*n* = 5 independent cultivation batches; *p* < 0.01), compared with the conventional single‐interface system. This enhancement arises from the expanded oxygen‐accessible area and is achieved without altering microbial strains or culture chemistry. The results demonstrate that a simple design of the growth environment can substantially improve fabrication efficiency while maintaining operational simplicity.

Beyond the high yield, control over bacterial localization enables spatial differentiation of the scaffold. Under the imposed oxygen gradients, bacteria preferentially accumulate at both parallel interfaces, where oxygen availability is highest. Concurrently, bacterial motility and continuous cellulose secretion in the aqueous phase promote the formation of an open fibrillar interlayer that forms out‐of‐plane, linking the two parallel biofilms. Confocal laser scanning microscopy (CLSM) of the as‐fabricated SDBC system, with cellulose stained using Calcofluor White, reveals a non‐uniform cross‐sectional distribution characterized by higher fibril density at the parallel regions (Figure 2c). Quantitative fluorescence analysis over increasing cultivation periods shows a progressive increase in cellulose content throughout the scaffold, with more pronounced accumulation at the parallel biofilms, consistent with sustained oxygen supply at A/W and W/O interfaces (Figure 2d). Scanning electron microscopy further corroborates the presence of the two dense surfaces (Figure 2e(i, iii)) connected by an intermediate region composed of a fibrillar interlayer (Figure 2e(ii)). These observations establish that oxygen availability regulates not only cellulose production but also the internal organization of the scaffold.

Because the spatially differentiated structure is formed during cultivation rather than through post‐growth processing, the environmental footprint of SDBC fabrication differs fundamentally from conventional BC biofilm production and downstream BC nanofiber processing. To assess whether spatial programming during growth offers environmental advantages relative to conventional static BC cultivation and subsequent top‐down fibrillation, a cradle‐to‐gate life cycle assessment (LCA) was conducted in accordance with ISO 14040 and ISO 14044 standards [32, 33]. Solvent recycling combined with a substantially higher production yield still leads to a 2% reduction in global warming potential (GWP) relative to the conventional static BC cultivation method during the biosynthesis and purification stage. In downstream processing, the elimination of mechanical fibrillation further reduces GWP by 54% relative to BC nanofiber production (Table S1–S4). Across seven TRACI v2.1 impact categories, the production of 1 kg SDBC exhibits a 22%–37% lower environmental burden than the conventional production of BC nanofibers (Figure 2f, g). As a result, SDBC exhibits a GWP of 115.9 kg CO_2_‐eq kg^−1^, compared with 148.5 kg CO_2_‐eq kg^−1^ calculated for conventional bacterial cellulose nanofibers (BCNF) using the same LCA framework. For broader context, Figure 2h also presents GWP values including 190 kg CO_2_‐eq kg^−1^ for wood‐pulp‐derived cellulose nanofibers (CNF) [34], 132.9 kg CO_2_‐eq kg^−1^ for sugarcane bagasse‐derived lignocellulosic nanofibers (LCNF) [35], and 261.8 kg CO_2_‐eq kg^−1^ for microcrystalline cellulose (MCC) [36]. Although differences in system boundaries and inventory assumptions should be considered, these values provide a broader context for the environmental profile of SDBC. By avoiding intensive post‐processing steps and associated energy and material inputs, this strategy provides a more resource‐efficient, sustainable pathway for developing structurally programmed cellulose‐based scaffolds.

### 2.2 Structural Characterization and Mechanical Evaluation of the SC Construct Upon Multicomponent Integration

**FIGURE 3:**
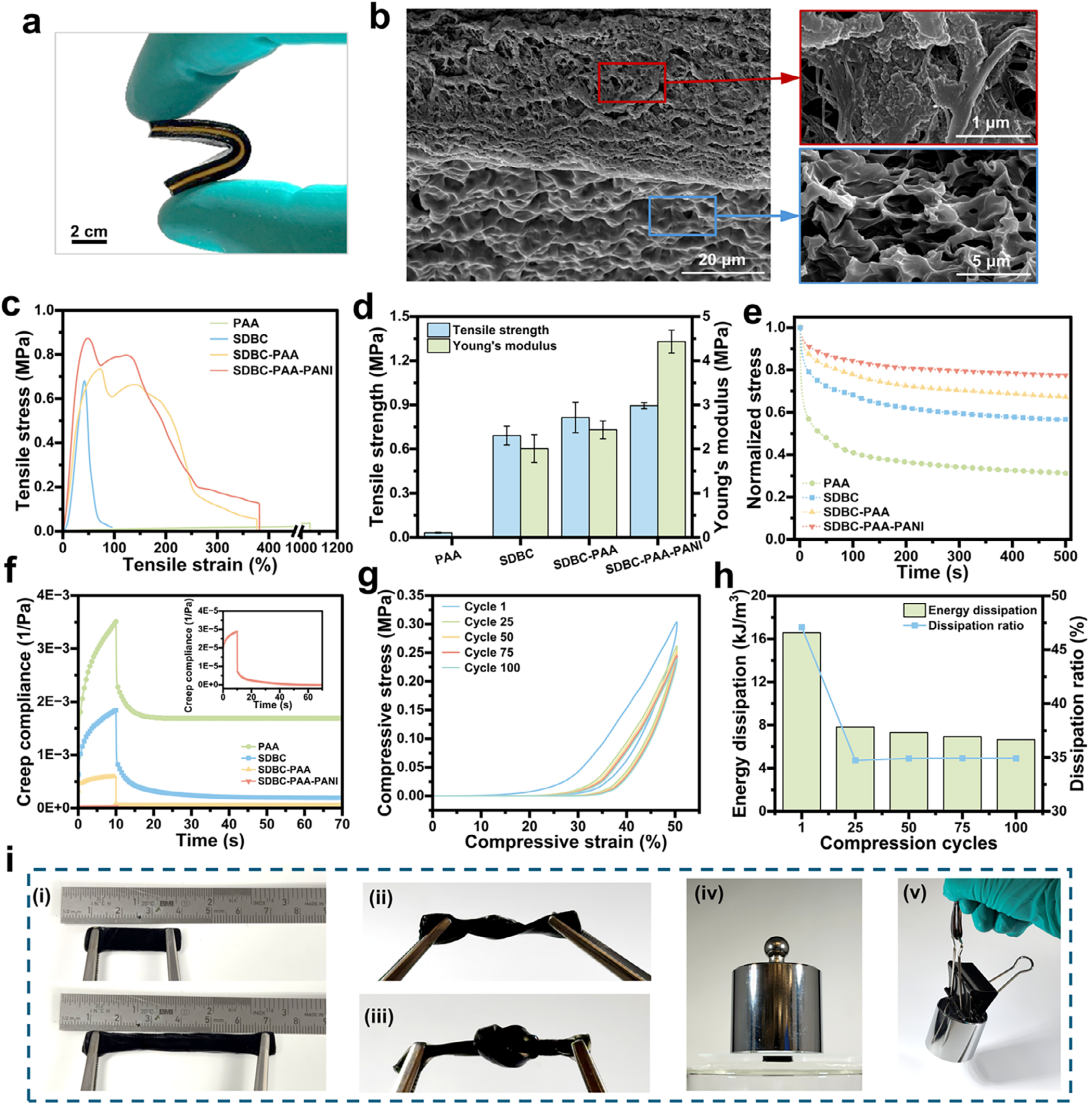
Structural characterization and mechanical performance of SDBC‐based composite hydrogels. (a) Side‐view photograph of SDBC‐PAA‐PANI hydrogel. (b) Scanning electron microscopy (SEM) images of the cross‐section of SDBC‐PAA‐PANI hydrogel, with higher‐magnification views highlighting the PANI‐rich electrode region outlined in red and the SDBC‐PAA electrolyte matrix outlined in blue. (c) Tensile stress‐strain curves of PAA, SDBC, SDBC‐PAA, and SDBC‐PAA‐PANI hydrogels. (d) Comparison of tensile strength and Young's modulus values of different hydrogel systems. (e) Stress relaxation behavior under constant strain. (f) Creep and recovery responses under constant stress followed by unloading. (g) Cyclic compression loading and unloading behavior of the SDBC‐PAA‐PANI hydrogel at 50% compressive strain. (h) Energy dissipation and dissipation ratio of the SDBC‐PAA‐PANI hydrogel during cyclic compression at 50% strain. (i) Photographs of SDBC‐PAA‐PANI hydrogels subjected to various deformation modes: (i) stretching, (ii) twisting, (iii) knotting, (iv) load bearing, and (v) lifting a 500 g weight.

The structural integrity of the SDBC architecture upon material loading was evaluated through a two‐step in situ polymerization process. First, poly (acrylic acid) (PAA) was generated within the SDBC matrix to form a solid‐state SDBC‐PAA electrolyte hydrogel, followed by conformal polymerization of aniline into an electroactive polyaniline (PANI) phase (Figure S1). An additional LCA of the complete SDBC‐PAA‐PANI device showed a GWP of 77.6 kg CO_2_‐eq kg^−1^ device. PANI synthesis was identified as the primary environmental hotspot, contributing 81.6% of the total GWP, followed by PAA synthesis (14.4%), BC culture (3.1%), and BC purification (0.9%) (Tables S5 and S6). The resulting SDBC‐PAA‐PANI construct is found to be mechanically flexible (Figure 3a), enabling facile handling and processing. Scanning electron microscopy (SEM) reveals the microstructural organization of the functionalized scaffold (Figure 3b). A more porous structure is observed at the interior of the SDBC‐PAA network, while denser regions near the interfaces are associated with PANI loading. High‐magnification imaging (Figure S2) and elemental mapping (Figure S3) confirm the loading of PANI evidenced by enrichment of nitrogen signals near the interfaces.

The strength and structural stability of the hydrogel system, reflecting the cooperative integration of the growth‐defined scaffold with the functional components (PAA and PANI), were systematically evaluated through tensile, rheology, and compression tests. The SDBC‐PAA‐PANI system showed a tensile strength of 0.89 MPa and a Young's modulus of 4.4 MPa upon axial loading along the plane (Figure 3c,d), which exceed those of the original SDBC (0.69 and 2.0 MPa, respectively) and PAA‐loaded SDBC (SDBC‐PAA, 0.81 and 2.4 MPa, respectively). Accordingly, the incorporation of PAA into SDBC substantially increases toughness, with SDBC‐PAA displaying a 5.7‐fold improvement relative to SDBC (Figure S4). This enhancement is further amplified upon PANI integration (SDBC‐PAA‐PANI), yielding a toughness of 1.77 MJ m^−3^, corresponding to a 7.7‐fold increase relative to SDBC. The pronounced energy dissipation observed during deformation may arise from network entanglement together with hydrogen bonding and electrostatic interactions among the components [13, 24, 37]. The molecular origin of these interactions is examined in detail in the following section.

Rheological measurements with oscillatory rotation applied parallel to the plane further elucidate the mechanical integrity of the composite network. Strain sweep tests show that all SDBC‐based hydrogel systems exhibit elastic‐dominated behavior, with storage modulus exceeding the loss modulus across the measured strain range (G' > G'') (Figure S5a). Successive incorporation of PAA and PANI results in marked increases in both moduli. Frequency sweep measurements reveal frequency‐independent behavior (Figure S5b), indicating a stable and well‐connected network, in agreement with the strong interchain interactions identified later at the molecular level.

The in‐plane resilience and recoverability were assessed using stress relaxation and creep‐recovery tests. In stress relaxation experiments conducted under constant strain (Figure 3e), SDBC‐PAA‐PANI exhibits the lowest relaxation rate at 22.5% (Figure S6), indicating superior resistance to structural rearrangement under sustained deformation. Consistent behavior is observed in creep‐recovery tests (Figure 3f), where samples were subjected to constant stress followed by unloading. Among all tested materials, SDBC‐PAA‐PANI displays the lowest relaxation and the highest recovery ratio (Figure S6), demonstrating structural stability and elastic recovery.

Compression tests were performed to further probe structural resilience against out‐of‐plane deformations. SDBC‐PAA‐PANI reaches a compressive strength of 0.60 MPa at 50% strain (Figure S7a). Progressive increases in compressive stress and hysteresis loop area with strain from 10% to 70% (Figure S7b) reflect sustained elastic behavior and effective energy dissipation. Anti‐fatigue performance was evaluated through cyclic compression loading and unloading (Figure 3g). SDBC‐PAA‐PANI exhibits a typical hysteresis loop with an initial energy dissipation during cyclic compression of 16.6 kJ m^−3^ at 50% strain in the first loading‐unloading cycle (Figure 3h), which decreases and stabilizes between cycles 2 and 25. Beyond 25 cycles, the hysteresis loops remain similar, with dissipated energy stabilizing at approximately 7 kJ m^−3^. The results indicate the attainment of a mechanically steady state and robust fatigue resistance under repeated deformation. The SDBC architecture provides a mechanically robust foundation for subsequent functionalization and electrochemical operation. The SDBC‐PAA‐PANI system, therefore, preserves flexibility while demonstrating stability and resiliency under various macroscopical deformation modes (Figure 3i). To elucidate the molecular interactions underlying this mechanical reinforcement, we next examine the chemical compatibility and interaction hierarchy within the system.

### 2.3 Multiscale Characterization of Chemical Interactions

**FIGURE 4:**
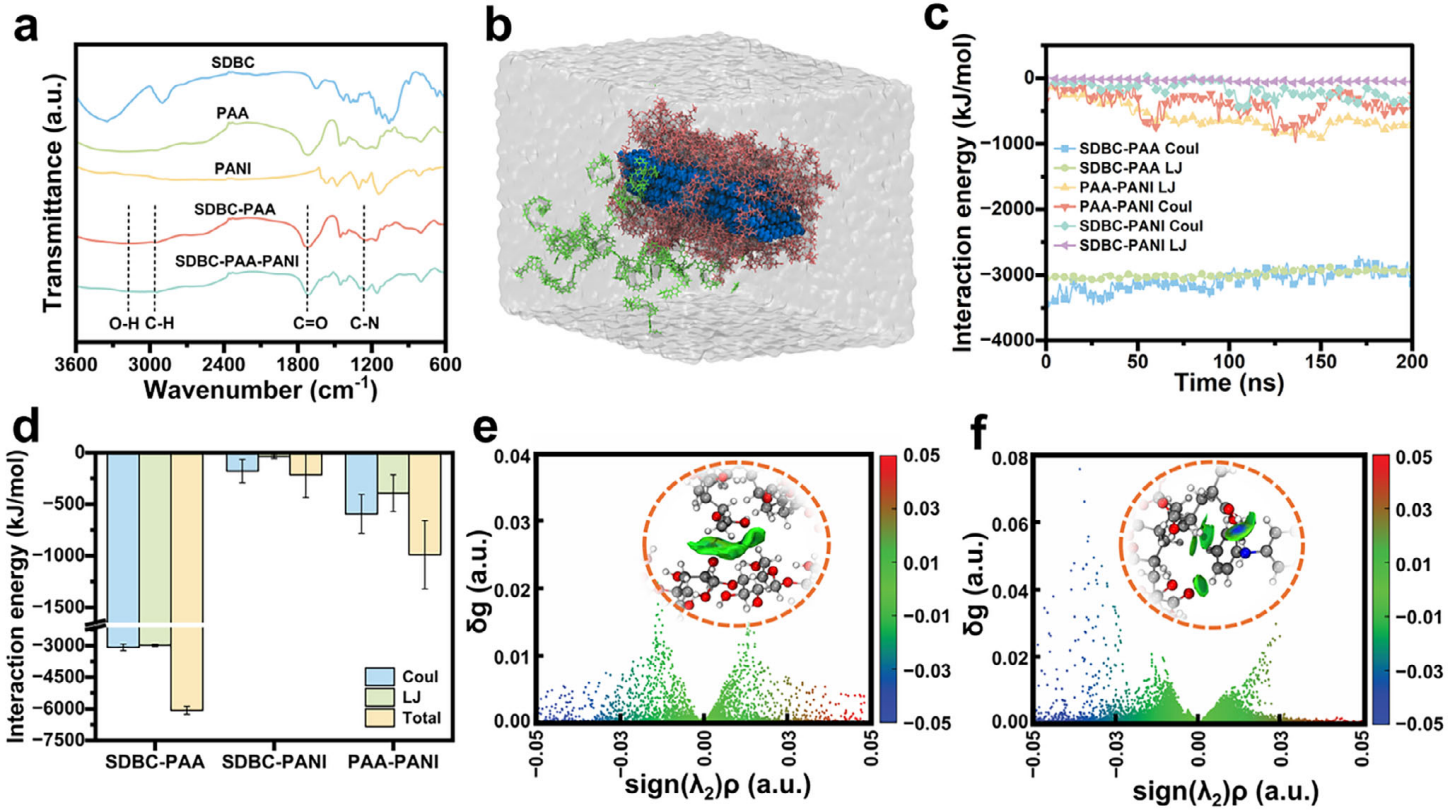
Chemical and molecular‐level characterization of the SDBC‐PAA‐PANI hydrogel. (a) Fourier transform infrared (FT‐IR) spectra of the SDBC, SDBC‐PAA, and SDBC‐PAA‐PANI composites. (b) Representative snapshot from molecular dynamics (MD) simulations of the SDBC‐PAA‐PANI hydrogel, where blue denotes SDBC, red denotes PAA, and green denotes PANI. (c) Interaction energy of SDBC‐PAA‐PANI hydrogel over 200 ns. (d) Time‐averaged interaction energies of the SDBC‐PAA‐PANI system over a 200 ns simulation. Independent Gradient Model (IGM) scatter plots and corresponding isosurfaces (isovalue = 0.005 a.u.) illustrating noncovalent interactions between SDBC and PAA (e) and between PAA and PANI (f).

Chemical interactions among SDBC, PAA, and PANI were examined to assess compatibility and intermolecular coupling within the composite. In the Fourier transform infrared (FT‐IR) spectra (Figure 4a), shifts in the bands assigned to C═O stretching (1719 cm^−1^) and O─H stretching (3175 cm^−1^), relative to the individual components, indicate hydrogen bonding between SDBC and PAA. Shifts in the C─N and O─H bands also suggest the enhanced O─H···N interactions between PANI and SDBC‐PAA. Molecular dynamics (MD) simulations were conducted to further elucidate the fundamental interactions governing composite compatibility. Inspection of simulation trajectories (Figure 4b) indicates strong SDBC affinity with PAA and PANI, consistent with thermodynamically favorable assembly. Quantitative analysis of interaction energies shows that the SDBC‐PAA pair exhibits strongly negative Coulombic (−3084 kJ mol^−1^) and Lennard–Jones (−2988 kJ mol^−1^) contributions (Figure 4c,d), reflecting pronounced electrostatic and van der Waals interactions. These interactions are supported by extensive interfacial contact (Figure S8) and hydrogen bonding (Figure S9). In addition, Independent Gradient Model (IGM) analysis shows a broad intermolecular interaction region between SDBC and PAA (Figure 4e). A more extensive interaction region occurs between the carboxyl groups of PAA and the amino groups of PANI (Figure 4f). Corresponding scatter plots show a more negative sign(λ_2_)ρ value of approximately −0.035 and a high δ value of approximately 0.078, confirming strong noncovalent interactions between PAA and PANI. The analyses clarify the interaction hierarchy within the system (Figure 1b). The SDBC is consolidated by hydrogen bonding between cellulose hydroxyl groups and PAA carboxyl groups, forming a mechanically robust framework. PAA serves as a mediator for PANI incorporation through hydrogen bonding. This dense network of noncovalent interactions underpins the high compatibility between the scaffold and functional components, providing a molecular basis for the mechanical integrity and coupled functional responses observed subsequently.

Beyond the evidence for extensive intermolecular coupling, Raman spectroscopy (Figure S10), X‐ray diffraction (XRD; Figure S11), and X‐ray photoelectron spectroscopy (XPS; Figure S12) provide complementary evidence for incorporation of PANI into the SDBC‐PAA scaffold. Deconvolution of the N 1s spectrum shows an N^+^/N ratio of 47.64% (Figure S13), confirming a substantial doping level of PANI. This result also indicates PANI predominantly adopts the electroactive emeraldine salt state [38].

### 2.4 Electrochemical Performances of SCs

**FIGURE 5:**
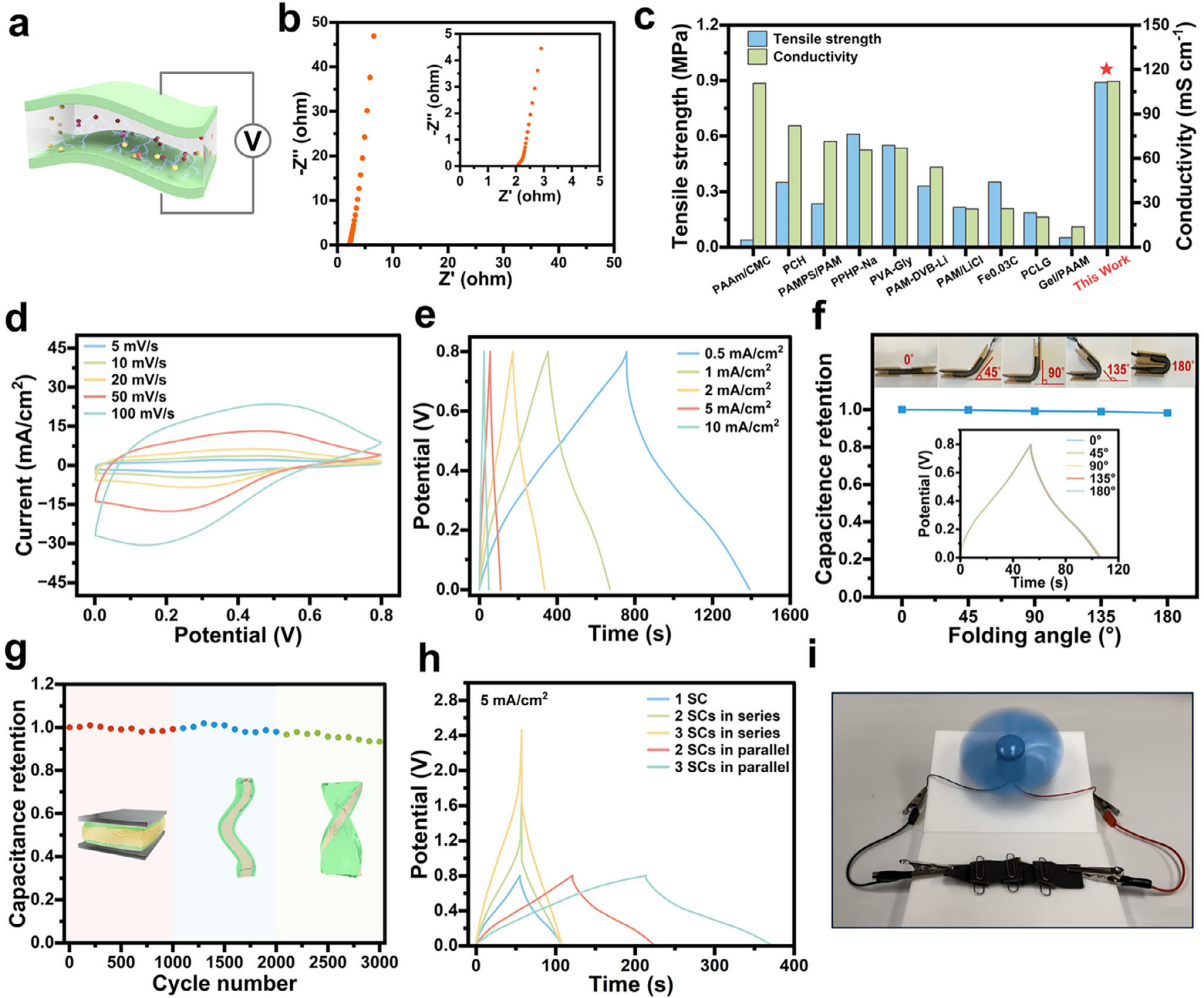
Electrochemical demonstration of transport in the SDBC‐PAA‐PANI system. (a) Schematic illustration of ion transport within the monolithic SDBC‐PAA‐PANI configuration. (b) Nyquist plot of SDBC‐PAA‐PANI. (c) Mechanical performance and ion conductivity of SDBC‐PAA‐PANI in comparison with other ion‐conductive hydrogels [3, 4, 8, 41, 43, 44, 45, 46, 47, 48]. (d) Cyclic voltammetry (CV) curves recorded at different scan rates. (e) Galvanostatic charge‐discharge (GCD) curves measured at various current densities. (f) Capacitance retention under different folding angles. (g) Capacitance retention under repeated compressing, bending, and twisting for 1000 cycles. (h) GCD curves of SDBC‐PAA‐PANIs connected in series and in parallel. (i) Photograph of three SDBC‐PAA‐PANI connected in series driving a fan.

Hierarchically organized electrochemical systems rely on balancing ion transport, active material loading, and structural stability [39]. The SDBC‐PAA‐PANI construct here provides a representative platform to probe ion transport, charge‐storage behavior, and structural stability within the spatially differentiated scaffold (Figure 5a).

To explore this structure‐transport relationship, electrochemical impedance spectroscopy was first employed to examine interfacial resistance and ion transport dynamics. Prior to testing, the aniline concentration was optimized, with 0.5 m selected to balance electroactive loading and structural integrity (Figure S14). A single‐layer BC scaffold produced at the air‐water interface under static cultivation was separately functionalized to form a BC‐PAA gel electrolyte and BC‐PAA‐PANI gel electrodes. These components were then assembled as a post‐assembled supercapacitor for comparison with the all‐in‐one SDBC‐PAA‐PANI device. The SDBC‐PAA‐PANI device showed a lower equivalent series resistance of 2.1 Ω cm^−2^ (Figure 5b) than the post‐assembled multilayer device (10.5 Ω cm^−2^; Figure S15), indicating more effective charge transport. The nearly vertical capacitive response in the low‐frequency region suggests minimal diffusion limitations and confirms efficient ion transport through the electrolyte domain [40]. These features are consistent with structural continuity of the scaffold, which minimizes contact resistance typically introduced by post‐assembly. Importantly, the observed transport behavior arises from pathways that are co‐formed during biosynthesis and span the scaffold without interruption, rather than from alignment or bonding of discrete layers. As a result, the SDBC‐PAA‐PANI device shows a high ionic conductivity of 112.0 mS cm^−1^ compared to the post‐assembled construct (23.8 mS cm^−1^), achieving a favorable balance between the ionic conductivity and mechanical strength (Figure 5c). Such high ionic conductivity facilitates rapid ion transport, which contributes to enhanced electrochemical performance during charge‐discharge processes. The same architectural principle may also benefit other soft electrochemical systems requiring continuous ion transport, including sensors and gel‐electrolyte devices.

SDBC‐PAA‐PANI was configured as an all‐in‐one supercapacitor to evaluate how the density‐differentiated architecture governs charge storage and transport behavior. Cyclic voltammetry (CV) curves (Figure 5d) recorded at scan rates from 5 to 100 mV s^−1^ exhibit well‐defined redox features of pseudocapacitive behavior associated with polyaniline [41]. Correspondingly, galvanostatic charge‐discharge (GCD) profiles (Figure 5e) maintain consistent shapes over a broad range of current densities, indicating reversible charge storage and stable electrode‐electrolyte coupling [42]. The areal capacitance reaches 410.6 mF cm^−2^ at 0.5 mA cm^−2^ and remains 325.9 mF cm^−2^ with a Coulombic efficiency of 97.9% at 10 mA cm^−2^ (Figure S16), reflecting efficient charge utilization and rate tolerance. As summarized in the Ragone plot (Figure S17), the device delivers a maximum areal energy density of 34.0 µWh cm^−2^ and retains 24.1 µWh cm^−2^ at a power density of 3644.2 µW cm^−2^. These values fall within the range reported for PANI‐based flexible and all‐in‐one supercapacitors (Table S7), confirming that the spatially differentiated scaffold supports competitive electrochemical performance while prioritizing mechanical robustness and stability. The overall electrochemical performance outperformed the post‐assembled control (Figure S15).

The electrochemical response can be directly correlated with the architecture of the system. The denser SDBC parallel biofilms provide abundant anchoring sites for PANI deposition, enabling a high electroactive mass loading of 6.8 mg cm^−2^ and supporting elevated capacitance values. This is consistent with previous studies which suggested that BC substrates with sufficient surface area could promote active material loading and charge carrier flow [9, 12]. In contrast, the lower‐density middle SDBC‐PAA region facilitates electrolyte uptake and ion migration, reducing transport resistance [13, 14]. Together, these features illustrate how growth‐defined density gradients enable concurrent mechanical reinforcement and transport functionality within a single structure.

The operational stability of the construct was evaluated under repeated electrochemical and mechanical deformations. When subjected to bending at different folding angles, including up to 180°, the GCD profiles of SDBC‐PAA‐PANI remain unchanged and capacitance retention exceeds 98% (Figure 5f). Additional tests involving repeated compression, bending, and twisting show capacitance retention above 90% after thousands of deformation cycles (Figure 5g), confirming that electrochemical functionality is preserved under sustained mechanical stress. During cyclic charge‐discharge testing at 5 mA cm^−2^, the capacitance retention remains at 87% after 5000 cycles with coulombic efficiency retained above 95.7%. Post‐cycling structural and XPS analyses further suggested that the structure and PANI chemical states were largely preserved (Figure S18), supporting its long‐term electrochemical stability.

To illustrate practical scalability, multiple SDBC‐PAA‐PANI units were connected in series and parallel to adjust output voltage and discharge duration (Figure 5h and Figure S19). As a simple demonstration, a series‐connected assembly was capable of powering small electronic components, including a miniature fan (Figure 5i and Video S1) and an LED light (Figure S20).

The electrochemical demonstration validates the ability of the growth‐defined SDBC to support efficient charge transport and mechanical resilience under functional loading. The results underscore the suitability of the biofabricated architecture as a general platform for layered and multifunctional material systems.

## 3 Conclusion

This work demonstrates a growth‐defined biosynthesis strategy for producing cellulose scaffolds with spatially differentiated density tailored to all‐in‐one supercapacitors. By engineering the oxygen environment during cultivation, an architecture is formed in which two parallel dense biofilms are interconnected by an interlayer fibrillar network in the out‐of‐plane direction of the scaffold. This growth‐defined structure provides a stable framework for subsequent functionalization while preserving structural continuity and enabling transport pathways that are encoded directly during biosynthesis rather than introduced through post‐growth lamination or assembly.

The scaffold's compatibility with functional components is demonstrated in an SDBC‐PAA‐PANI all‐in‐one supercapacitor. In this context, in situ incorporation of poly (acrylic acid) and the polyaniline electroactive phases results in strong intermolecular interactions and coherent integration throughout the scaffold. The resulting composite exhibits enhanced mechanical properties, including a tensile strength of 0.89 MPa and a Young's modulus of 4.4 MPa, as well as stable performance under repeated deformation. The SDBC system supports electroactive material at the parallel, dense interfaces while enabling ion transport through the fibrillar interlayer, validating the functional relevance of the spatial differentiation.

Broadly, this one‐step bottom‐up biosynthesis circumvents extensive post‐processing and enables direct encoding of structural continuity and functional gradients during growth. Future studies examining oxygen distribution, bacterial localization, regional porosity, and interfacial mechanical properties will help clarify how oxygen‐dependent bacterial behavior will affect scaffold formation and device performance. By enabling differentiated soft material architectures in which transport pathways emerge during growth from controlled biological assembly, this approach establishes a versatile and sustainable fabrication route to develop cellulose scaffolds that can serve as mechanically robust platforms for multifunctional soft electrochemical systems in which transport and mechanical function emerge from the biosynthesized architecture.

## 4 Experimental Section

### 4.1 Materials

Peptone, yeast extract, sodium phosphate dibasic, D‐(+)‐Glucose, acrylic acid (AA), α‐ketoglutaric acid (α‐KG), N, N’‐methylene bisacrylamide (MBA), aniline (ANI) monomer, ammonium persulfate (APS), hydrochloric acid, and sulfuric acid were purchased from Sigma–Aldrich. Citric acid and sodium hydroxide pellets were obtained from Fisher Chemical. 3 m Novec HFE‐7500 was purchased from TMC Industries, Inc. (USA). All reagents were employed without further purification. Milli‐Q (IQ 7000, WP‐300‐1) was used throughout the experiments.

### 4.2 SDBC Cultivation

Hestrin‐Schramm (HS) medium was prepared by dissolving glucose (20 g L^−1^), Na_2_HPO_4_ (2.5 g L^−1^), yeast extract (5 g L^−1^), and peptone (5 g L^−1^) in Milli‐Q water, with the pH adjusted to 4.5 using citric acid. *Komagataeibacter medellinensis* was inoculated into the freshly prepared HS medium at a concentration of 2% (v/v) and transferred into the PTFE mould, prior to which, a layer of dense HFE‐7500 oil was placed at the bottom of the culture medium. Cultivation was carried out at room temperature (23°C) to minimize oil evaporation. The bacteria were able to proliferate and produce cellulose nanofibers with oxygen supplied simultaneously through the air‐water (A/W) and water‐oil (W/O) interfaces. The SDBC was harvested after certain cultivation periods and purified with 0.1 m NaOH at 80°C for 2 h to remove any residues. The SDBC samples were subsequently rinsed thoroughly with Milli‐Q water for at least three cycles (24 h) and stored at 4°C for further steps and analysis. All glassware, moulds, culture media, and pipette tips were sterilized by autoclaving at 121°C for 20 min. The non‐autoclavable HFE oil was sterilized by filtration through 0.2 µm syringe filters (Whatman Puradisc 30, sterile).

### 4.3 Fabrication of the SDBC‐PAA Binary Composite

A precursor solution was prepared by dissolving acrylic acid (AA, 20 wt.%), MBA crosslinker (0.1 wt.% relative to AA), and photo initiator (0.4 wt.% relative to AA) in 50 mL of Milli‐Q water. The solution was purged with nitrogen for 20 min to remove dissolved oxygen. Prior to impregnation, the SDBC hydrogel was gently and uniformly compressed to a predefined thickness of ∼3 mm to standardize its water content. The hydrogel was then immersed in the precursor solution overnight (8 h) to allow complete monomer penetration. After soaking, the hydrogel was placed in the mould (20 × 20 × 3 mm) and gently covered to remove excess solution, ensuring controlled water content and monomer loading. Photopolymerization was subsequently performed under UV irradiation (365 nm, 0.15 W cm^−2^) for 30 min, resulting in the formation of SDBC‐PAA composite.

### 4.4 Development of SDBC‐PAA‐PANI Ternary Composite

SDBC‐PAA‐PANI was prepared by in situ polymerization of ANI on both surfaces of SDBC. ANI monomer was added to hydrochloric acid solution (1.0 m, 50 mL) to form solution A, while APS was dissolved in hydrochloric acid (1.0 m, 50 mL) to form solution B. The SDBC‐PAA composite was pre‐soaked in the solution A for 1 h, followed by the addition of solution B to initiate polymerization (ANI/APS = 1:1). The PANI loading was controlled by varying the ANI concentration (0.3–0.7 m). The reaction was carried out at 0°C–5°C for 6 h. Afterward, the samples were thoroughly washed with deionized water to remove residual monomers and impurities.

### 4.5 Statistical Analysis

All measurements were conducted in triplicate unless otherwise stated (*n* ≥ 3). Mean values and standard deviations were calculated using IBM SPSS Statistics 27. Statistical significance was assessed using one‐way analysis of variance (ANOVA), with *p* < 0.05 considered statistically significant. Data visualization was performed using Origin (version 2024).

## Author Contributions

**Xin Shu**: conceptualization, investigation, validation, methodology, visualization, formal analysis, writing – original draft, writing – review and editing. **Yi Lu**: conceptualization, methodology, validation, supervision, visualization, writing – review and editing. **Xuetong Shi**: data curation, investigation, methodology, validation. **Zhangmin Wan**: methodology, investigation, validation, data curation, software. **Peijin Jiang**: methodology, validation, investigation, data curation. **Minke Yang**: investigation, validation. **Qingshi Tu**: methodology, validation, data curation. **Orlando J. Rojas**: conceptualization, validation, supervision, project administration, writing – review and editing, funding acquistion, resources.

## Conflicts of Interest

The authors declare no conflicts of interest.

## Supporting information




**Supporting File 1**: advs77603‐sup‐0001‐SuppMat.docx.

**Supporting File 2**: advs77603‐sup‐0002‐VideoS1.mp4.

## Data Availability

The data that support the findings of this study are available from the corresponding author upon reasonable request.
