# Supplementary material for: Biosynthesis of Spatially Differentiated Cellulose for Flexible All‐in‐one Supercapacitors
Source: Adv Sci (Weinh). 2026 Sep 27:e77603. Online ahead of print. doi: 10.1002/advs.77603 (PMC13616339; doi:10.1002/advs.77603)
Supplement: Supplementary file 1 [file ADVS-9999-e77603-s001.docx]

Supporting Information

**Biosynthesis of Spatially Differentiated Cellulose for Flexible All-in-one Supercapacitors**

*Xin Shu ^a,b^, Yi Lu ^b,c,*^, Xuetong Shi ^a,b^, Zhangmin Wan ^a,b^,* *Peijin Jiang ^b,d^, Minke Yang ^b,d^, Qingshi Tu ^b,d^, Orlando J. Rojas ^a,b,d,e,*^*

*^a^* Department of Chemical and Biological Engineering, The University of British Columbia, 2360 East Mall, Vancouver, BC V6T 1Z3, Canada

*^b^* Bioproducts Institute, The University of British Columbia, 2385 East Mall, Vancouver, BC V6T 1Z4, Canada

*^c^* Institute of Process Engineering, Chinese Academy of Sciences, Beijing 100190, PR China

*^d^* Department of Wood Science, The University of British Columbia, 2424 Main Mall, Vancouver, BC V6T 1Z4, Canada

*^e^* Department of Chemistry, The University of British Columbia, 2036 Main Mall, Vancouver, BC V6T 1Z1, Canada

*^*^* **Authors for Correspondence:** Yi Lu: luyi@ipe.ac.cn; Orlando J. Rojas: orlando.rojas@ubc.ca

**Experimental section**

**Structural characterization**

The SDBC hydrogel was stained with Calcofluor White and was examined using Confocal Laser Scanning Microscopy (CLSM, Leica Stellaris 5) equipped with an inverted microscope (4×/NA 0.16, UPLSAPO). Images were acquired with under 405 nm laser excitation, and fluorescence emission was collected in the range of 425–530 nm. The morphologies of gel samples were observed under Scanning Electron Microscopy (SEM, Helios NanoLab 650) with an acceleration of 10.0 kV. Fourier-Transform Infrared (FT-IR) spectra were measured using an IR spectrophotometer Spectrum 100 (Bruker), in the range of 400 to 4000 cm^−1^ (resolution 4 cm^−1^, 32 scans). Raman spectra were recorded on a Horiba LabRAM HR Evolution using a 532 nm laser, with the scanning range from 500–3000 cm^−1^. X-ray photoelectron spectroscopy (XPS) was performed using an electron spectrometer (Thermo Scientific K-Alpha). X-ray diffraction (XRD) patterns of the samples were collected using a Bruker D8-Advance X-ray diffractometer (Copper Kα1 & Kα2 radiation, λ = 1.5406 Å) in the 2*θ* range of 5°–80° with 0.5° min^−1^ rate and 0.02° step size.

**Life cycle assessment**

*Goal and scope definition.* To assess the environmental impacts of spatially differentiated BC scaffold (SDBC) production and SDBC-PAA-PANI device, a cradle-to-gate life cycle assessment (LCA) was conducted. The functional unit was defined as 1 kg dry weight. For comparison, a conventional BC biofilm and BC nanofiber production were also modelled. Environmental impacts were evaluated using the TRACI v2.1 method across seven impact categories, including acidification (AP), climate change (GWP), ecotoxicity: freshwater (EC), eutrophication (EP), human toxicity: carcinogenic (HTP-c), human toxicity: non-carcinogenic (HTP-nc), and particulate matter formation (PMF). The LCA model did not consider the manufacturing of equipment, as its normalized environmental impacts were found to be insignificant.

*Life cycle inventory.* The life cycle inventory (LCI) for SDBC, conventional BC biofilm, BC nanofiber, and SDBC-PAA-PANI device was developed based on data obtained from laboratory experiments. LCI modelling was performed using the Ecoinvent v3.10 database with the cut-off system model. All processes were assumed to be in British Columbia, Canada, and the corresponding regional electricity mix was applied. Details regarding the calculation of electricity consumption and the selection of proxy flows adopted when specific inventory data were unavailable in the database are described in the table notes.

**Mechanical properties**

The tensile properties of the hydrogels (10 × 5 mm) were evaluated using an Instron 3345 materials testing system equipped with a 500 N load cell. The displacement speed was set as 0.5 mm s^−1^. Compression tests were conducted under different strain levels and loading-unloading cycles at a displacement rate of 5 mm min^−1^. The viscoelastic behavior of hydrogels was examined using an Anton Paar rheometer (MCR 302e) with a 25 mm parallel plate. For the study of creep recovery behaviors, samples were given a constant stress (10 Pa) for 10 s (creep), and zero stress for 60 s (recovery). The compliance (Pa^−1^) value was monitored along the process. The stress-relaxation behaviors were measured when samples were subjected to a constant strain (0.05%) for 500 s. The evolution of stress as a function of holding time was recorded. Strain sweep tests were measured in the range of the 0.01%–100% at 1 Hz, and the frequency sweep was performed from 0.01–10 Hz at 0.1% strain.

**MD simulation**

Cellulose Iα crystallites, consisting of an 18-chain model, were constructed based on the atomic structure obtained from high-resolution X-ray diffraction data using the Nanopolysaccharide Builder (NPB) program [1]. Polymeric chains, including 100 PAA chain (10 repeat units, 722.7 Da, 100% protonation, neutral termini, and zero net charge) and 20 polyaniline chains (8 repeat units, 674.6 Da, 50% protonation degree based on experimental results), were generated using the CHARMM-GUI Ligand Reader and Modeler module [2]. All atomistic molecular dynamics (MD) simulations were performed with NAMD version 3.0, employing the CHARMM36 force field for cellulose [3]. The CHARMM General Force Field (CGenFF) for PAA and PANI [4]. and the TIP3P water model [5]. The composite system was assembled using Packmol [6] with 1 M NaCl added to achieve charge neutrality. The fixed protonation states, as in related materials simulations, represent electrostatic screening and charge compensation [7-8].

The modeled compositions were as follows:

| System | Cellulose  chains | PANI  chains | PAA  chains | Water | Na^+^ back-ground | Total Cl- | Net e^-^ charge |
| --- | --- | --- | --- | --- | --- | --- | --- |
| Cellulose/  PANI | 18 | 20 | 0 | 45,746 | 20 (~0.02 M) | 100 (20 background + 80 counterions) | 0 |
| Cellulose/  PANI/PAA | 18 | 20 | 100 | 46,369 | 20 (~0.02 M) | 100 (20 background + 80 counterions) | 0 |

A preliminary equilibration run was carried out in the NPT ensemble at 303.15 K and 1 atm pressure environment, with temperature and pressure regulated using Langevin dynamics [9] and the Langevin piston method [10], respectively. This 1-ns equilibration ensured adequate solvent relaxation and removal of any steric overlaps. The subsequent production simulations were extended for 100 ns under the same ensemble conditions. Long-range electrostatic interactions were computed using the particle-mesh Ewald (PME) method, and a spherical cutoff of 12 Å was applied to truncate short-range van der Waals and electrostatic interactions. All covalent bonds involving hydrogen atoms were constrained using the SHAKE/RATTLE algorithms [11].

Hydrogen-bond and energy decomposition analyses were conducted using the *gmx hbond* and *gmx energy* utilities in GROMACS version 2018.6 [12]. with a geometric hydrogen-bonding criterion of 3.5 Å and 30°. The inter-molecular noncovalent interaction visualizations were analyzed using the Independent Gradient Model (IGM) method [13]. applied to the final MD snapshot and computed with the Multiwfn package (version 3.8 dev) [14]. The number of atomic contacts between cellulose and other polymeric chains was quantified using a custom Tcl script in VMD. All molecular visualizations were prepared with VMD version 1.9.3 [15].

**Electrochemical performances**

Before electrochemical characterization, the device was activated by cyclic voltammetry for 50 cycles at a scan rate of 50 mV s^−1^ until the CV response stabilized. For the electrochemical tests, SDBC-PAA-PANI (10 × 10 × 3 mm) were soaked in 1M sulfuric acid solution and sandwiched between two pieces of carbon cloth, where the carbon cloth served as current collector. The electrochemical properties of SDBC-PAA-PANI were investigated using a two-electrode configuration on an electrochemical workstation (WaveDriver^®^ 200, Pine Research Instrumentation, Inc., USA). Cyclic voltammetry and galvanostatic charge-discharge measurements were performed within a voltage window of 0–0.8 V over a range of scan rates and current densities. Electrochemical impedance spectroscopy was carried out from 0.01 Hz to 100 kHz with the potential amplitude of 5 mV. Deformation cycles were performed without any holding period. A compressive strain of 50% was applied at a crosshead speed of 5 mm min^−1^. For bending, the clamp separation was reduced from 20 to 10 mm, then returned to its initial position at 5 mm s^−1^. The device was twisted from 0° to 180° and then returned to 0° at an angular velocity of 90° s^−1^. Electrochemical performance was measured after unloading and at each 100-cycle interval.

The area-specific capacitance (C_a_, mF cm^−2^) of the supercapacitors was calculated from the GCD profiles according to eq (1):

$C_{a}=\frac{I\times\Delta t}{a\times\Delta U}$ (1)

where I (mA) denote the discharge current, Δt (s)is the discharge time, a (cm^2^) represents the working area of the supercapacitor, and ΔU (V) is voltage excluding IR drop.

The areal energy density E_a_ (µWh cm^−2^) and power density P_a_ (µW cm^−2^) were subsequently calculated using the corresponding eq (2) and eq (3):

$E_{a}=\frac{C_{a}\times\Delta U^{2}}{2\times3.6}$	(2)

$P_{a}=\frac{E_{a}\times3600}{\Delta t}$	(3)

The ionic conductivity δ (mS cm^−1^) was calculated according to the eq (4):

$\delta=\frac{l}{R\times a}$	(4)

where l (cm) and a (cm^2^) are the thickness and working area of the supercapacitors. The resistance R (Ω) was the intercept on the real axis of the Nyquist plot obtained from EIS measurements.

Figure S1. Surface SEM images of a) SDBC, b) SDBC-PAA, and c) SDBC-PAA-PANI.

Figure S2. The cross-section image of SDBC-PAA-PANI under an optical microscope.

Figure S3. SEM image of the cross-section of the SDBC-PAA-PANI and its corresponding mapping of elements. Scale bar is 200 µm.

Figure S4. Toughness of PAA, SDBC, SDBC-PAA, and SDBC-PAA-PANI.

Figure S5. Storage modulus (G') and loss modulus (G'') of PAA, SDBC, SDBC-PAA, and SDBC-PAA-PANI under oscillatory strain sweep (a) and frequency sweep (b).

Figure S6. Creep recovery and relaxation rate of PAA, SDBC, SDBC-PAA, and SDBC-PAA-PANI.

Figure S7. a) Compressive stress-strain curves of PAA, SDBC, SDBC-PAA, and SDBC-PAA-PANI at 50% strain. b) The continuous loading-unloading compressive cyclic curves of SDBC-PAA-PANI.

Figure S8. a) Atom number of PAA and PANI located within 10 Å of the SDBC surface over 200 ns. b) Average atom number of PAA and PANI located within 10 Å of the SDBC surface.

Figure S9. Number of hydrogen bonds among SDBC-PAA, SDBC-PANI, and PAA-PANI.

Figure S10. Raman spectra of SDBC-PAA, PANI, and SDBC-PAA-PANI.

Figure S11. XRD patterns of SDBC-PAA, PANI, and SDBC-PAA-PANI.

Figure S12. a) XPS survey and b) high-resolution C 1s spectra of SDBC-PAA and SDBC-PAA-PANI.

Figure S13. High-resolution N 1s X-ray photoelectron spectroscopy (XPS) spectrum of SDBC-PAA-PANI.

Figure S14. a) Areal capacitance, b) Ragone plot, and c) Nyquist plots of supercapacitors with different aniline concentrations.

Figure S15. Electrochemical performance of the post-assembled BC-PAA-PANI device. a) Nyquist plot. b) Cyclic voltammetry (CV) curves at different scan rates. c) Galvanostatic charge-discharge (GCD) curves at different current densities. d) Areal capacitance and Coulombic efficiency calculated from the GCD curves at different current densities. e) Ragone plot of areal energy density versus areal power density.

Figure S16. Areal capacitance and Coulombic efficiency of SDBC-PAA-PANI at different current densities calculated from GCD curves.

Figure S17. Ragone plot showing areal energy density versus areal power density of the SDBC-PAA-PANI, presented in the context of reported PANI-based flexible and all-in-one supercapacitors [16-24].

Figure S18. a) Capacitance retention rate and coulombic efficiency during 5000 charge/discharge cycles. b) Nyquist plot of SDBC-PAA-PANI after 5000 charge/discharge cycles. c) Scanning electron microscopy (SEM) images of the cross-section of SDBC-PAA-PANI hydrogel after 5000 charge/discharge cycles, with higher-magnification views highlighting the PANI-rich electrode region outlined in red and the SDBC-PAA electrolyte matrix outlined in blue. d) High-resolution N 1s X-ray photoelectron spectroscopy (XPS) spectrum of SDBC-PAA-PANI after 5000 charge/discharge cycles.

Figure S19. CV curves of supercapacitors in series and parallel.

Figure S20. Three supercapacitors connected in series lighting up a LED bulb.

Table S1. Life cycle inventory of producing 1kg SDBC via multiphasic fabrication route

| **Process** | **Input/Output** | **Flow** | **Amount** | **Unit** |
| --- | --- | --- | --- | --- |
| **BC culture** | Input | Sodium phosphate | 1.25 | kg |
|  |  | Protein^1^ | 2.50 | kg |
|  |  | Fodder yeast | 2.50 | kg |
|  |  | Citric acid | 1.50 | kg |
|  |  | Glucose | 10.00 | kg |
|  |  | Tap water | 500.00 | kg |
|  |  | Electricity, medium voltage ^2^ | 233.00 | kWh |
|  |  | Solvent, organic ^3^ | 32.20 | kg |
|  | Output | Culture medium | 500.00 | L |
| **BC purification** | Input | Culture medium | 500.00 | L |
|  |  | Tap water ^4^ | 2000.00 | kg |
|  |  | Sodium hydroxide | 4.00 | kg |
|  |  | Electricity, medium voltage ^5^ | 221 | kWh |
|  | Output | SDBC ^6^ | 1.00 | kg |
|  |  | Wastewater, average | 2.504 | m^3^ |

^1^ Proxy for peptone.

^2^ Electricity for sterilizing culture medium was estimated from laboratory autoclave operation (1.4 kW, 1.5 h per cycle, 4.5 L per cycle), corresponding to 0.46 kWh L⁻¹. Sterilization of 500 L therefore required approximately 233 kWh.

^3^ Proxy for HFE 7500 fluids. For each 100 mL of culture medium, 200 mL of HFE-7500 fluid was used and recycled. Assuming a 2% loss during recycling, the total loss was 20 L (32.2 kg).

^4^ 2,000 kg of water: 1,000 kg for NaOH preparation and 1,000 kg for washing.

^5^ Electricity consumption for NaOH solution preparation was estimated by accounting for hot-water generation using an electric kettle and subsequent heating on a hot plate. The kettle contributed 0.264 kWh per 4 L of hot water, while heating 4 L of 0.1 M NaOH on a hot plate consumed 0.62 kWh, assuming 50% of rated power. Based on these values, preparing 1,000 L of NaOH solution required a total of 221 kWh, with 66 kWh attributed to kettle use and 155 kWh to hot plate heating.

^6^ Based on a yield of 0.2 g dry weight obtained per 100 mL of culture medium from experiments. The cellulose yield was measured following the method reported in our previous study [25]. The average yield value obtained from five independent cultivation batches collected on day 10 was used as the corresponding inputs for the LCA assessment of the multiphasic cultivation system.

Table S2. Life cycle inventory of producing 1kg BC biofilm and BC nanofiber via conventional static cultivation route

| **Process** | **Input/Output** | **Flow** | **Amount** | **Unit** |
| --- | --- | --- | --- | --- |
| BC culture | Input | Sodium phosphate | 1.92 | kg |
|  |  | Fodder yeast | 3.85 | kg |
|  |  | Citric acid | 3.85 | kg |
|  |  | Protein | 2.31 | kg |
|  |  | Glucose | 15.38 | kg |
|  |  | Tap water | 769.23 | kg |
|  |  | Electricity, medium voltage | 358.98 | kWh |
|  | Output | Culture medium | 769.23 | L |
| BC purification | Input | Culture medium | 769.23 | L |
|  |  | Tap water | 3076.92 | kg |
|  |  | Sodium hydroxide | 6.15 | kg |
|  |  | Electricity, medium voltage | 340 | kWh |
|  | Output | Purified BC biofilm ^1^ | 1.00 | kg |
|  |  | Wastewater, average | 3.85 | m^3^ |
| BC fibrillation | Input | Electricity, medium voltage ^2^ | 225.00 | kWh |
|  |  | Purified BC biofilm | 1.00 | kg |
|  | Output | BC nanofiber | 1.00 | kg |

^1^ Based on experimental results, a yield of 0.13 g dry weight per 100 mL of culture medium was obtained. The cellulose yield was measured following the method reported in our previous study [25]. The average yield value obtained from five independent cultivation batches collected on day 10 was used as the corresponding inputs for the LCA assessment of the conventional BC cultivation system.

^2^ Electricity consumption for mechanical nanofibrillation of BC was estimated for 1 kg dry mass at 1.0 wt% solids (100 L suspension). The process comprised blending and microfluidization. Blending (1.5 kW, 30 min per 2 L batch) required 50 batches, corresponding to 37.5 kWh. Microfluidization (1.5 kW, 4 L h⁻¹) required 25 h to process 100 L, consuming an additional 37.5 kWh. As the nanofibrillation sequence was repeated three times, the total electricity demand was estimated to be 225 kWh per kg of dry BC.

Table S3. LCA results of producing 1kg SDBC via multiphasic fabrication route

|  | **Unit** | **BC culture** | **BC purification** | **Total** |
| --- | --- | --- | --- | --- |
| **Acidification** | kg SO_2_-Eq | 4.31E-01 | 8.04E-02 | 5.12E-01 |
| **Climate change** | kg CO_2_-Eq | 9.01E+01 | 2.58E+01 | 1.16E+02 |
| **Ecotoxicity: freshwater** | CTUe | 2.23E+03 | 6.89E+02 | 2.92E+03 |
| **Eutrophication** | kg N-Eq | 4.43E-01 | 1.98E-01 | 6.41E-01 |
| **Human toxicity: carcinogenic** | CTUh | 3.42E-05 | 1.27E-05 | 4.69E-05 |
| **Human toxicity: non-carcinogenic** | CTUh | 2.60E-05 | 1.70E-05 | 4.30E-05 |
| **Particulate matter formation** | kg PM_2.5_-Eq | 7.99E-02 | 3.75E-02 | 1.17E-01 |

Table S4. LCA results of producing 1kg BC biofilm and BC nanofiber via conventional static cultivation route

|  | Unit | BC  culture | BC purification | BC  fibrillation | Total |
| --- | --- | --- | --- | --- | --- |
| Acidification | kg SO_2_-Eq | 5.13E-01 | 1.24E-01 | 3.70E-02 | 6.73E-01 |
| Climate change | kg CO_2_-Eq | 9.22E+01 | 3.97E+01 | 1.65E+01 | 1.48E+02 |
| Ecotoxicity: freshwater | CTUe | 2.74E+03 | 1.06E+03 | 4.62E+02 | 4.26E+03 |
| Eutrophication | kg N-Eq | 5.43E-01 | 3.05E-01 | 7.32E-02 | 9.22E-01 |
| Human toxicity: carcinogenic | CTUh | 3.82E-05 | 1.95E-05 | 5.28E-06 | 6.30E-05 |
| Human toxicity: non-carcinogenic | CTUh | 3.26E-05 | 2.61E-05 | 5.12E-06 | 6.38E-05 |
| Particulate matter formation | kg PM_2.5_-Eq | 1.03E-01 | 5.76E-02 | 2.64E-02 | 1.87E-01 |

Table S5. Life cycle inventory of producing 1kg dry weight SDBC-PAA-PANI device using SDBC

| **Process** | **Input/Output** | **Flow** | **Amount** | **Unit** |
| --- | --- | --- | --- | --- |
| **BC culture** | Input | Sodium phosphate | 0.034 | kg |
|  |  | Protein | 0.067 | kg |
|  |  | Fodder yeast | 0.067 | kg |
|  |  | Citric acid | 0.040 | kg |
|  |  | Glucose | 0.269 | kg |
|  |  | Tap water | 13.459 | kg |
|  |  | Electricity, medium voltage | 6.272 | kWh |
|  |  | Solvent, organic | 0.867 | kg |
|  | Output | Culture medium | 13.459 | L |
| **BC purification** | Input | Culture medium | 13.459 | L |
|  |  | Tap water | 53.836 | kg |
|  |  | Sodium hydroxide | 0.108 | kg |
|  |  | Electricity, medium voltage | 5.949 | kWh |
|  | Output | SDBC | 26.918 | g |
|  |  | Wastewater, average | 0.067 | m^3^ |
| **PAA synthesis** | Input | Electricity, medium voltage ^1^ | 119.785 | kWh |
|  |  | SDBC | 26.918 | g |
|  |  | Acrylic acid | 628.533 | g |
|  |  | Chemical, organic ^2^ | 3.096 | g |
|  | Output | BC-PAA hydrogel | 658.143 | g |
| **PANI synthesis** | Input | BC-PAA hydrogel | 658.143 | g |
|  |  | Aniline | 6729.475 | g |
|  |  | Ammonium Sulfate ^3^ | 16487.214 | g |
|  |  | Hydrochloric Acid | 4759.085 | g |
|  |  | Tap Water | 139.973 | kg |
|  | Output | SDBC-PAA-PANI device | 1.000 | kg |
|  |  | Wastewater | 0.168 | m^3^ |

^1^ Proxy for α-Ketoglutaric acid and N, N’-methylene bisacrylamide.

^2^ Electricity consumption for PAA synthesis was estimated based on the irradiation time with a 50 W UV light source.

^3^ Proxy for ammonium persulfate.

Table S6. LCA results of producing 1kg dry weight SDBC-PAA-PANI device using SDBC

|  | Unit | BC  culture | BC purification | PAA synthesis | PANI synthesis | Total |
| --- | --- | --- | --- | --- | --- | --- |
| Acidification | kg SO_2_-Eq | 1.16E-02 | 2.16E-03 | 2.83E-02 | 4.24E-01 | 4.66E-01 |
| Climate change | kg CO_2_-Eq | 2.43E+00 | 6.95E-01 | 1.11E+01 | 6.33E+01 | 7.76E+01 |
| Ecotoxicity: freshwater | CTUe | 6.00E+01 | 1.85E+01 | 2.73E+02 | 3.69E+03 | 4.04E+03 |
| Eutrophication | kg N-Eq | 1.19E-02 | 5.34E-03 | 5.82E-02 | 2.79E-01 | 3.55E-01 |
| Human toxicity: carcinogenic | CTUh | 9.20E-07 | 3.42E-07 | 3.27E-06 | 1.93E-05 | 2.38E-05 |
| Human toxicity: non-carcinogenic | CTUh | 7.01E-07 | 4.58E-07 | 4.40E-06 | 7.21E-05 | 7.77E-05 |
| Particulate matter formation | kg PM_2.5_-Eq | 2.15E-03 | 1.01E-03 | 1.54E-02 | 4.91E-02 | 6.77E-02 |

Table S7. Electrochemical and mechanical performances of the current work and the recently reported all-in-one supercapacitor device.

| **Material** | **Tensile strength (MPa)** | **Capacitance^a^**  **(mF cm^–2^)** | **Ionic conductivity (mS cm^−1^)** | **Energy density**  **(μWh cm^–2^)** | **Power density**  **(μW cm^–2^)** | **Reference** |
| --- | --- | --- | --- | --- | --- | --- |
| This work | 0.89 | 410.6 at 0.5 mA cm^−2^ | 112.0 | 34.0 | 3644.2 | This work |
| PCLG | 0.19 | 45 at 0.2 mA cm^–2^ | 20.3 | 15.85 | 132.03 | [16] |
| PAM-DVB-Li | 0.33 | 469 at 0.5 mA cm^−2^ | 54 | 31.9 | 1325 | [17] |
| PAM/LiCl | 0.22 | 364.0 at 0.3 mA cm^−2^ | 25.7 | 16.4 | 880.8 | [18] |
| PCH | 0.35 | 244 at 0.2 mA cm^−2^ | 82 | 21 | 800 | [19] |
| PVA/PHEA | 1.07 | 98 at 0.2 mA cm^−2^ | 85 | 8.48 | 78.52 | [20] |
| PPHP-Na | 0.61 | 250.8 at 0.5 mA cm^−2^ | 65.6 | 34.8 | 250 | [21] |
| (β)CD-g-Cell | - | 501.8 at 1.0 mA cm^−2^ | - | 44.6 | 400.00 | [22] |
| PAD | 1.8 | 430 at 0.5 mA cm^–2^ | 57 | 22 | 150 | [23] |
| P(AM-AA)/PVA | - | 95.8 at 0.2 mA cm^–2^ | - | 8.5 | 80 | [24] |
| Cellulose/PAM | 0.05 | 417.5 at 1.0 mA cm^−2^ | - | 37.11 | 400 | [26] |
| PVA-AA | - | 48.1 at 1.0 mA cm^–2^ | - | 4.3 × 10^−3^ | 19.3 | [27] |
| PGS | 0.55 | 238 at 0.2 mA cm^−2^ | 66.9 | 16.5 | 50 | [28] |
| PVA-PA | - | 212.5 at 0.5 mA cm^−2^ | 52.6 | 18.89 | 197.8 | [29] |
| PAMPS/PAM | 0.023 | 441 at 1.0 mA cm^–2^ | 71.3-171.0 | 157.97 | 762.30 | [30] |
| PACP/PVA | - | 317 at 1 mA cm^−2^ | - | 27.6 | 2500 | [31] |
| Gel/PAAM/PPy | 0.027 | 363.8 at 0.5 mA cm^–2^ | 30.7 | 32.33 | 400 | [32] |

^a^ All capacitance values are compared on a device-level basis.

**References**

[1]	Z. Wan, Y. Yu, G. F. M. Bautista, S. Srebnik, J. C. Smith, O. J. Rojas, “Nanopolysaccharide Builder: A User-Friendly Tool for Atomistic Models of Polysaccharide-Based Nanostructures,” *Journal of Chemical Information and Modeling* **2025**, *65* (21), https://doi.org/10.1021/acs.jcim.5c01204.

[2]	S. Jo, T. Kim, V. G. Iyer, W. Im, “CHARMM-GUI: A web-based graphical user interface for CHARMM,” *Journal of Computational Chemistry* **2008**, *29* (11), 1859, https://doi.org/10.1002/jcc.20945.

[3]	O. Guvench, S. S. Mallajosyula, E. P. Raman, E. Hatcher, K. Vanommeslaeghe, T. J. Foster, F. W. Jamison, II, A. D. MacKerell, Jr., “CHARMM Additive All-Atom Force Field for Carbohydrate Derivatives and Its Utility in Polysaccharide and Carbohydrate–Protein Modeling,” *Journal of Chemical Theory and Computation* **2011**, *7* (10), 3162, https://doi.org/10.1021/ct200328p.

[4]	K. Vanommeslaeghe, A. D. MacKerell, Jr., “Automation of the CHARMM General Force Field (CGenFF) I: Bond Perception and Atom Typing,” *Journal of Chemical Information and Modeling* **2012**, *52* (12), 3144, https://doi.org/10.1021/ci300363c.

[5]	P. Mark, L. Nilsson, “Structure and Dynamics of the TIP3P, SPC, and SPC/E Water Models at 298 K,” *The Journal of Physical Chemistry A* **2001**, *105* (43), 9954, https://doi.org/10.1021/jp003020w.

[6]	L. Martínez, R. Andrade, E. G. Birgin, J. M. Martínez, “PACKMOL: A package for building initial configurations for molecular dynamics simulations,” *Journal of Computational Chemistry* **2009**, *30* (13), 2157, https://doi.org/10.1002/jcc.21224.

[7]	F. S. Emami, V. Puddu, R. J. Berry, V. Varshney, S. V. Patwardhan, C. C. Perry, H. Heinz, “Force Field and a Surface Model Database for Silica to Simulate Interfacial Properties in Atomic Resolution,” *Chemistry of Materials* **2014**, *26* (8), 2647, https://doi.org/10.1021/cm500365c.

[8]	T.-J. Lin, H. Heinz, “Accurate Force Field Parameters and pH Resolved Surface Models for Hydroxyapatite to Understand Structure, Mechanics, Hydration, and Biological Interfaces,” *The Journal of Physical Chemistry C* **2016**, *120* (9), 4975, https://doi.org/10.1021/acs.jpcc.5b12504.

[9]	G. E. Uhlenbeck, L. S. Ornstein, “On the Theory of the Brownian Motion,” *Physical Review* **1930**, *36* (5), 823, https://doi.org/10.1103/PhysRev.36.823.

[10]	S. E. Feller, Y. H. Zhang, R. W. Pastor, B. R. Brooks, “Constant pressure molecular dynamics simulation: The Langevin piston method,” *Journal of Chemical Physics* **1995**, *103* (11), 4613, https://doi.org/10.1063/1.470648.

[11]	H. C. Andersen, “Rattle: A “velocity” version of the shake algorithm for molecular dynamics calculations,” *Journal of Computational Physics* **1983**, *52* (1), 24, https://doi.org/10.1016/0021-9991(83)90014-1.

[12]	M. J. Abraham, T. Murtola, R. Schulz, S. Páll, J. C. Smith, B. Hess, E. Lindahl, “GROMACS: High performance molecular simulations through multi-level parallelism from laptops to supercomputers,” *SoftwareX* **2015**, *1-2*, 19, https://doi.org/10.1016/j.softx.2015.06.001.

[13]	T. Lu, Q. Chen, “Independent gradient model based on Hirshfeld partition: A new method for visual study of interactions in chemical systems,” *Journal of Computational Chemistry* **2022**, *43* (8), 539, https://doi.org/10.1002/jcc.26812.

[14]	T. Lu, “A comprehensive electron wavefunction analysis toolbox for chemists, Multiwfn,” *Journal of Chemical Physics* **2024**, *161* (8), https://doi.org/10.1063/5.0216272.

[15]	W. Humphrey, A. Dalke, K. Schulten, “VMD: Visual molecular dynamics,” *Journal of Molecular Graphics* **1996**, *14* (1), 33, https://doi.org/10.1016/0263-7855(96)00018-5.

[16]	Z. Li, H. Zhang, C. Li, X. Tian, S. Liu, G. Qin, J. Yang, Q. Chen, “Extreme condition-tolerant stretchable flexible supercapacitor and triboelectric nanogenerator based on carrageenan-enhanced gel for energy storage, energy collection and self-powered sensing,” *International Journal of Biological Macromolecules* **2024**, *273*, 132994, https://doi.org/10.1016/j.ijbiomac.2024.132994.

[17]	Y. Li, X. Wei, F. Jiang, Y. Wang, M. Xie, J. Peng, C. Yi, J. Li, M. Zhai, “An Ultrastretchable and Highly Conductive Hydrogel Electrolyte for All-in-One Flexible Supercapacitor With Extreme Tensile Resistance,” *Energy & Environmental Materials* **2025**, *8* (2), e12820, https://doi.org/10.1002/eem2.12820.

[18]	D. Wang, F. Yang, C. Wang, F. Chu, J. Nan, R. Chen, “In-situ polymerization of PANI on hydrogel electrolyte enabling all-in-one supercapacitors mechanically stable at low temperatures,” *Chemical Engineering Journal* **2023**, *455*, 140949, https://doi.org/10.1016/j.cej.2022.140949.

[19]	K. Wang, X. Zhang, C. Li, X. Sun, Q. Meng, Y. Ma, Z. Wei, “Chemically Crosslinked Hydrogel Film Leads to Integrated Flexible Supercapacitors with Superior Performance,” *Advanced Materials* **2015**, *27* (45), 7451, https://doi.org/10.1002/adma.201503543.

[20]	J. Yang, X. Yu, X. Sun, Q. Kang, L. Zhu, G. Qin, A. Zhou, G. Sun, Q. Chen, “Polyaniline-Decorated Supramolecular Hydrogel with Tough, Fatigue-Resistant, and Self-Healable Performances for All-In-One Flexible Supercapacitors,” *ACS Applied Materials & Interfaces* **2020**, *12* (8), 9736, https://doi.org/10.1021/acsami.9b20573.

[21]	R. Wang, D. Lei, H. Zhang, J. Liang, Z. Shang, L. Wan, Z. Li, C. Si, “An interface-integrated hydrogel for all-in-one flexible supercapacitor with excellent wide-temperature and self-healing properties,” *Composites Part B: Engineering* **2024**, *275*, 111345, https://doi.org/10.1016/j.compositesb.2024.111345.

[22]	Q. Gong, Y. Li, X. Liu, Z. Xia, Y. Yang, “A facile preparation of polyaniline/cellulose hydrogels for all-in-one flexible supercapacitor with remarkable enhanced performance,” *Carbohydrate Polymers* **2020**, *245*, 116611, https://doi.org/10.1016/j.carbpol.2020.116611.

[23]	Y. Shi, Y. Zhang, L. Jia, Q. Zhang, X. Xu, “Stretchable and Self-Healing Integrated All-Gel-State Supercapacitors Enabled by a Notch-Insensitive Supramolecular Hydrogel Electrolyte,” *ACS Applied Materials & Interfaces* **2018**, *10* (42), 36028, https://doi.org/10.1021/acsami.8b13947.

[24]	J. Huang, S. Han, J. Zhu, Q. Wu, H. Chen, A. Chen, J. Zhang, B. Huang, X. Yang, L. Guan, “Mechanically Stable All Flexible Supercapacitors with Fracture and Fatigue Resistance under Harsh Temperatures,” *Advanced Functional Materials* **2022**, *32* (35), 2205708, https://doi.org/10.1002/adfm.202205708.

[25]	Y. Lu, Y. Chun, X. Shi, D. Wang, F. Ahmadijokani, O. J. Rojas, “Multiphase Under-Liquid Biofabrication With Living Soft Matter: A Route to Customize Functional Architectures With Microbial Nanocellulose,” *Advanced Materials* **2024**, *36* (27), 2400311, https://doi.org/10.1002/adma.202400311.

[26]	Y. Li, Q. Gong, X. Liu, Z. Xia, Y. Yang, C. Chen, C. Qian, “Wide temperature-tolerant polyaniline/cellulose/polyacrylamide hydrogels for high-performance supercapacitors and motion sensors,” *Carbohydrate Polymers* **2021**, *267*, 118207, https://doi.org/10.1016/j.carbpol.2021.118207.

[27]	J. Zhu, S. Han, Q. Wu, J. Zhang, A. Chen, B. Huang, J. Huang, L. Guan, “All-in-One Configured Flexible Supercapacitor for Wide-Temperature Operation and Integrated Application,” *ACS Applied Energy Materials* **2023**, *6* (8), 4157, https://doi.org/10.1021/acsaem.2c03800.

[28]	K. Zhu, X. Han, S. Ye, P. Cui, L. Dou, W. Ma, S. Heng, X. Tao, X. Wei, “Flexible all-in-one supercapacitors enabled by self-healing and anti-freezing polymer hydrogel electrolyte,” *Journal of Energy Storage* **2022**, *53*, 105096, https://doi.org/10.1016/j.est.2022.105096.

[29]	Y. Zhang, X. Wang, M. Yu, M. K. Hadi, S. Zhou, Y. Wang, F. Ran, “Heparin Doped Polyaniline for Anticoagulation Supercapacitors,” *Advanced Healthcare Materials* **2025**, *14* (13), 2500493, https://doi.org/10.1002/adhm.202500493.

[30]	J. Yang, M. Guo, L. Feng, J. Hao, Y. Guo, Z. Li, S. Liu, G. Qin, G. Sun, Q. Chen, “Swelling-resistant microgel-reinforced hydrogel polymer electrolytes for flexible all-in-one supercapacitors with high performances,” *Journal of Materials Chemistry C* **2023**, *11* (22), 7419, https://doi.org/10.1039/D3TC00160A.

[31]	X. Tao, Y. Wang, W. Ma, S. Ye, K. Zhu, L. Guo, H. Fan, Z. Liu, Y. Zhu, X. Wei, “Copolymer hydrogel as self-standing electrode for high performance all-hydrogel-state supercapacitor,” *Journal of Materials Science* **2021**, *56* (28), 16028, https://doi.org/10.1007/s10853-021-06304-3.

[32]	Y. Li, X. Liu, Y. Yang, C. Qian, C. Chen, L. Han, Q. Han, “A stretchable and self-healable conductive hydrogels based on gelation/polyacrylamide/polypyrrole for all-in-one flexible supercapacitors with high capacitance,” *Colloids and Surfaces A: Physicochemical and Engineering Aspects* **2022**, *636*, 128145, https://doi.org/10.1016/j.colsurfa.2021.128145.
